# Implant-based Prepectoral Breast Reconstruction: The Importance of Oncoplastic Plane, its Blood Supply and Assessment Methods

**DOI:** 10.29252/wjps.10.1.108

**Published:** 2021-01

**Authors:** Sharat Chopra, Robert D. Rehnke, Vidya Raghavan

**Affiliations:** 1 Cardiff & Vale University Health Board, Breast Centre, University Hospital Llandough, Penlan Road, Cardiff, Wales, United Kingdom;; 2 6606 10th Avenue N, St. Petersburg, Fla. USA;; 3 New Cross Hospital, Wolverhampton Road, Wolverhampton, West Midlands, United Kingdom.

**Keywords:** Breast, Anatomy, Prepectoral, Reconstruction, Vascularity, Skin flaps

## Abstract

With the recent rise in prepectoral breast reconstruction, partly due to the improvement in implants and the aesthetic results but, more so, due to the perseveration of the pectoralis major, it is of great importance to have an appreciation of the clinical anatomy with regards to the breast and its blood supply for the practice of prepectoral breast reconstruction. The preservation of the mastectomy flap vasculature together with meticulous surgical technique minimizes complications, notoriously of skin flap necrosis. We aimed to describe the anatomy of the oncoplastic plane (for the prepectoral technique), its vasculature, and relevant assessment methods.

## INTRODUCTION

The treatment of breast cancer has evolved from radical to more preservative oncological surgery with recent advances in implant-based reconstruction and biomaterials. These have led to increases in both classical techniques (e.g., subpectoral breast reconstruction) and more novel techniques such as prepectoral (muscle-sparing) reconstruction. The latter becoming increasingly popular since the pectoralis major is preserved, so shoulder dysfunction is avoided. Indeed, better understanding of the anatomy of the breast and its vasculature is of great importance when performing minimally invasive surgery and may well limit the incidence of skin flap necrosis (5%-30%)^1^. We aim to describe the anatomy of the oncoplastic plane, its vasculature and relevant assessment methods.

**Anatomy of the breast**

 The breast is a subcutaneous organ that normally extends antero-posteriorly between the second and the sixth ribs and mediolaterally between the sternum and the anterior axillary line ^2^. It rests on the fascia over the chest wall muscles (pectoralis major posteriorly, external oblique aponeurosis inferiorly, and serratus anterior laterally). Anteriorly, the skin is lined by subcutaneous fat, separating the superficial fascia.

The axillary tail extends laterally towards the axilla. Indeed, the shape, contour, size, and density vary amongst individuals. 

The breast consists of adipose, glandular, and fibro-glandular tissues. The fibro-glandular tissue (breast parenchyma) is composed of 15–20 lobes, subdivided into 20–40 lobules, which in turn consist of 10–100 alveoli. Fibrous bands of connective tissue, called the suspensory ligaments (the eponymous ligaments of Cooper) emerge from the superficial fascia to the skin^2^. 

**Fig.1:**
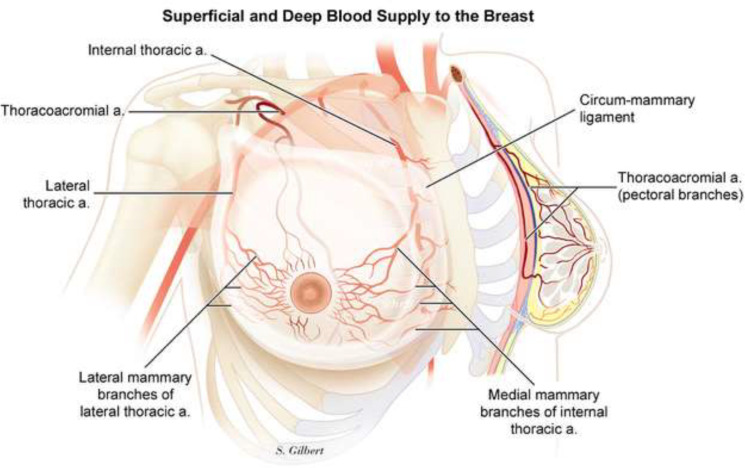
Superficial and deep blood supply of the breast (Copyright is observed)

The superficial and deep blood supply to the breast (Fig. 1) is through three main sources: i) medially located internal mammary perforators (approximately 60% of the blood supply); ii) branches of the lateral thoracic artery (approximately 30%); and iii) minor contributions from the thoracoacromial, intercostals, subscapular, and thoracodorsal arteries^2^^,^^3^. The perforating vessels form a subdermal plexus, which mainly supplies the breast skin. 

**The plane of dissection**

The prepectoral space has been defined as the potential space between the breast skin flap and the pectoralis fascia and the muscle^4^. Therefore, a good prepectoral space with well vascularized skin flaps are essential for this technique for which a good understanding of the plane of dissection is required to achieve success. The principle of this plane is based on the normal oncological plane which aims to preserve the vascularity of the mastectomy flaps while ensuring no breast tissue is not left behind^5^. Less than 0.02% of breast glandular tissue is present in this region^6^. 

The subdermal fat plane (between the sub-dermis and breast parenchyma) is a suitable guide for mastectomy. The subcutaneous layer lies between the dermis and breast tissue but is noted to be of variable thickness (0.5 to 2 cm) in different quadrants of the breast; it is a layer that is often dissected by many surgeons. Wide variability in the thickness of skin flap and presence of fascial plane has been observed, ranging from 0 to 29 mm in patients undergoing reduction mammoplasty (n=38)^7^. Therefore, a single uniform mastectomy plane cannot be obtained nor dissected.

Normally the subdermal fascial plane is adopted, and it is rarely more than 2 cm in thickness. However recently, anterior and posterior laminae have been described covering the main gland. Hence dissection over the corpus mammary gland with preservation of anterior lamina fat under the dermis would enable to preserve the vascularity of the mastectomy skin flap with minimal thickness of at least 8 mm would minimize the complications of mastectomy skin flap necrosis^8^.

**Fig. 2:**
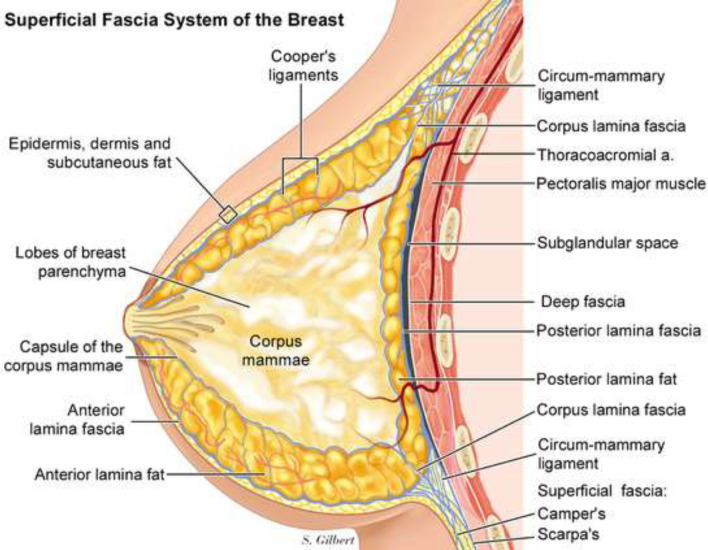
Superficial fascial system- sagittal view (Copyright is observed)

The senior author believes^9^ that by noting two key principles: firstly, the thickness of the skin and superficial fascial system(SFS) envelope not only varies from person to person but is thickest at the chest wall/circus-mammary ligament (Fig. 2), and tapers to its thinnest portion at the areolar border, where it is a couple of millimeters. Secondly, the perforators from the internal mammary and lateral mammary system, as well as the 4th and 5th anterior intercostals, travel through the adhesion ring of the superficial fascia to the deep fascia that surrounds the breast - the circum-mammary ligament. If one separates the capsule of the corpus mammae from the circum-mammary ligament, instead of cutting through it. Then you preserve these vessels of the superficial system of circulation to the skin envelope and Nipple. They run deep to the anterior lamina superficial fascia and superficial to the corpus mammae, so dissection at the capsule of the corpus mammae preserves these vessels.

**Methods of assessment of flap vascularity**

A compromise to the blood supply of the skin flaps may result in skin necrosis, poor tissue healing, infection and can even lead to Implant extrusion/failure. As such, appropriate assessment of the skin flap vascularity is imperative to the success of the operation and can be conducted through either a systematic clinical assessment or by using special devices to look for adequate flap vascularity intraoperatively. A clinical approach has been described by the senior author elsewhere^4^.

An ideal patient selection^10^ is by far the most crucial step in considering a prepectoral implant reconstruction. Preoperative assessment of patient skin quality including elasticity, previous radiotherapy to the skin and other negatively impacting factors such as high BMI, diabetes mellitus, or current smoking status adversely impacts the outcome. Sometimes, it may be possible to assess the breast skin thickness preoperatively by using digital mammography^11^ or magnetic resonance Imaging (MRI)^12^ which can aid to the suitability of this procedure. Oncological factors should also be taken into account before contemplating prepectoral breast reconstruction which includes a) proximity of the tumor to the chest wall or pectoralis fascia, i.e. in those patients where tumor is close to the chest wall or pectoral fascia there is a high risk for local recurrence thus may not be suitable candidate to prepectoral breast reconstruction, b) patients with aggressive cancer presentation such as inflammatory type or with fixed axillary nodes should not be considered for upfront reconstruction. 

Intraoperative flap assessment^13^^,^^14^ is done by visual inspection and palpation of the mastectomy flap thickness, non-exposure of the dermis during dissection, good capillary refill and bleeding skin edges all of which indicate adequate flap vascularity. Role of intraoperative blood/ tissue pH assessment for flaps has been studied with variable outcomes^15^^-^^17^. 

Special tissue perfusion assessment techniques/ devices have been used at various centers which most commonly uses Indocyanine green angiography with real-time arterial and venous flow measurement before and after prosthesis placement to assess flap vascularity in equivocal cases. With further advancement, a Laser-assisted Indocyanine Green fluorescent angiography (SPY) system has come into use which can check the viability of the flaps with higher sensitivity and specificity^18^^-^^20^.

Incisions placement over the mastectomy flaps need a special mention, as it is imperative to avoid incisions that can compromise flap vascularity and ultimately cosmetic outcome although keeping oncological safety at the forefront. Incisions placed on the lateral aspect or in the inframammary fold can avoid complications^21^. Incisions placed around the Nipple should best be avoided in order not to jeopardize the blood supply.

**Tackling a compromised Flap**

Even though, with all the above precautions to minimize the risk of vascular compromise to the flap, we might occasionally encounter some patients with poor vascularity of the flap. 

The author has developed a grading system for assessment of the mastectomy flap vascularity described previously^4^. Addressing this in several ways, firstly, by converting a prepectoral reconstruction to a subpectoral reconstruction, secondly, instead of using a fixed volume implant or fully inflated prosthesis one can deflate the prosthesis or use a prosthesis with minimal inflation for at least 3 wk before starting inflation so the skin flaps can vascularise^22^ and are in no undue tension or a delayed reconstruction altogether lastly, in patients with large ptotic breasts where the flaps are long and preservation of the nipple-areolar complex can be difficult perhaps, use of either, a reduction pattern or a simultaneous mastopexy^23^ with minimal underlying skin tension could reduce the subsequent risk of flap necrosis.

Postoperative assessment of reconstruction^24^^,^^25^, care of reconstruction in the form of avoidance of infection, identification or monitoring for partial or complete thickness tissue loss, use of local vasodilator ointments or creams^26^ to minimize congestion to the skin is vital. Special wound dressings such as honey-based^27^, silicone tapes or non-adherent skin dressing may be useful in preventing thickened scars and promote wound healing could be employed.

Use of closed incision negative pressure dressings^28^ has been suggested in breast reconstructive surgery has shown to be well-tolerated, adaptable, and reliable dressing capable of reducing postsurgical complications and improving scar outcomes in patients presenting with high-risk factors. 

## CONCLUSION

Mastectomy skin flap necrosis is a significant complication in breast cancer treatment; with the development of pre-pectoral implant-based reconstructions, the vascularity of the mastectomy skin ﬂap is the key to ensuring a successful outcome. However, for this to occur, the subcutaneous layer must be preserved appropriately and be of adequate thickness to minimize complications such as skin necrosis. Whilst there are promising modalities for assessing modalities, further prospective work is required before their use can become widespread. 

## CONFLICT OF INTEREST

The authors declare that there is no conflict of interests.
